# A Novel Approach of Periodontal Osseous Wall Piezosplitting and Sequential Bone Expansion in Management of Localized Intra-Bony Defects with Wide Angulation—A Randomized Controlled Trial

**DOI:** 10.3390/healthcare11060791

**Published:** 2023-03-08

**Authors:** Mahmoud Taha El-Destawy, Mohamed Fekry Khedr, Mostafa Mohamed Hosny, Ahmed Mohamed Bilal, Ahmed Mohamed Elshamy, Ibrahim Sabry El sayed, Abd el-latif galal Borhamy, Abd al-aziz kamal Aboamo, Ahmed Yousef Gamal

**Affiliations:** 1 Department of Oral Medicine and Periodontology and Diagnosis and Oral Radiology, Faculty of Dental Medicine, Al-Azhar University, Cairo 11651, Egypt; 2 Department of Periodontology, Faculty of Dental Medicine, Ain Shams University, Cairo 11566, Egypt

**Keywords:** bone swaging, periodontal regeneration, ethylenediaminetetraacetic acid, intra-bony pocket, wound healing

## Abstract

Piezoelectric surgical instruments with various mini-sized tips and cutting technology offer a precise and thin cutting line that could allow the wider use of periodontal osseous wall swaging. This randomized controlled trial was designed to investigate the use of a minimally invasive piezo knife to harvest vascularized interseptal bone pedicles in treating intra-bony defects. Sixteen non-smoking patients (mean age 39.6 ± 3.9) with severe chronic periodontitis were randomly assigned into one of two groups (N = 8). The Group 1 (control) patients were treated by bone substitute grafting of the intra-bony defect, whereas the Group 2 patients were treated by intra-bony defect osseous wall swaging (OWS) combined with xenograft filling of the space created by bone tilting. In both groups, the root surfaces were treated with a neutral 24% EDTA gel followed by saline irrigation. Clinical and radiographic measurements were obtained at baseline and 6 months after surgery. The sites treated with osseous wall swaging showed a statistically significant probing-depth reduction and increase in clinical attachment compared with those of the Group 1 patients. The defect base level was significantly reduced for the OWS group compared to that of the Group 1 control. By contrast, the crestal bone level was significantly higher in the OWS group compared to Group 1. The crestal interseptal bone width was significantly higher in Group 2 at 6 months compared to the baseline value and to that of Group 1 (<0.001). The osseous wall swaging effectively improved the clinical hard- and soft-tissue parameters. The use of mini inserts piezo-cutting, sequential bone expanders for osseous wall redirection, and root surface EDTA etching appears to be a reliable approach that could allow the use of OWS at any interproximal dimension.

## 1. Introduction

Treatment outcomes following periodontal therapy, especially closed mechanical debridement, access flap procedures and bone grafting usually take place through the long junctional epithelium, connective tissue attachment, root ankylosis, or unpredictable amounts of true regeneration at the most coronal part of the defect [1,2,3]. The multifaceted and complex process of periodontal regeneration entails the repair of the cementum, periodontal ligament, and alveolar bone [4]. However, complete true periodontal regeneration is an uncommon finding, even when using regenerative materials, such as guided tissue membranes and/or different biologics [5]. The most important factor that affects the outcome of therapy is defect angulation, which is the angle between the bony walls of the pocket and the root surface [6,7]. Usually, the most apical and middle parts of the defect possess narrower angulation compared to the most coronal third of the defect, a factor which could explain the incomplete healing of wide periodontal defects or the most coronal third of intra-bony defects following the application of most current regenerative approaches. Many studies reported the limits of narrow and wide defect angulations and their influence on treatment outcomes [8,9]. Most of these studies reported more significant bone gain with narrow angulations compared to wide angulations following open flap debridement, guided tissue membranes, or Emdogain therapy. Cortellini and Tonetti [10] defined ≤25 degrees as a narrow angulation and ≥37 degrees as wider. They reported that defects with narrow angulation are less liable to recur, in a long-term follow-up. Gamal et al. [11] failed to demonstrate any significant outcomes following the treatment of wide periodontal defects with PRF or PRGF compared to open flap debridement.

A wide defect angulation was recently reported to be associated with significant clot retraction within the defect, with an associated wide gap between the root surface and the clotted blood. This gap is responsible for the root-surface epithelialization and bacterial colonization that occurs following open flap debridement as a single therapy for wide defects [12]. Gap distance also could act as a dead space within which oozing clotted blood serum could accumulate and act as a good medium for bacterial colonization [13]. Gamal et al. [12] performed an experimental trial to measure the dimensions of the clot shrinkage within the intra-bony defects of 40-degree angulation. They reported blood clot shrinkage of about 1% following open flap debridement or bone grafting. They also reported that EDTA root surface treatment with or without bone grafting enhances blood clot adsorption to the root surface, a factor that could minimize gap dimensions and subsequently enhance regeneration. Many treatment options have been developed with the side effect of improving blood clot–root surface adhesion, such as growth factor application [14], root surface bio-modification [15], platelet concentrate preparations [11], enamel matrix derivatives [16], and laser therapy [17]. However, the outcomes of most of these options are limited, mainly because of the open contaminated nature of periodontal defects and the wide defect angulation.

Bone swaging is a technique that was used for the immediate reduction in periodontal defect angulation [18]. Bone swaging requires an edentulous area adjacent to the defect from which bone is pushed into contact with the root surface without fracturing the bone at its base [19]. Due to difficulties in accessibility the interdental bony walls, the use of bone swaging is limited to defects with adjacent edentulous areas or wide interdental defects of more than 4 mm [20]. Although bone swaging procedures offer the advantages of minimal surgical invasion and the inherent osteogenic properties of the vital bone [21], clinical investigations, applicability, and procedure guidelines are still limited. Piezoelectric surgical instruments, with their various mini-sized tips and cutting technology, offer a precise and thin cutting line that could allow the wider use of periodontal osseous wall swaging.

This randomized, prospective, single-centered, parallel clinical study aimed to assess the use of a minimally invasive piezo knife to harvest vascularized interseptal bone pedicles in treating intra-bony defects after an observation period of 6 months.

## 2. Materials and Methods

### 2.1. Ethical Consideration and Patient Recruitment

The study was conducted in accordance with the Declaration of Helsinki and approved by the Institutional Review Board of Al-Azhar University, Cairo, Egypt (“#839/2757”) for studies involving humans. Research procedures were explained to all patients, who agreed to participate in the study and signed the appropriate informed consent form of Al-Azhar University. This clinical trial is registered at national clinical trial registry (NCT03670979).

Sixteen non-smoking patients aged 28 to 51 years (mean age 39.6 ± 3.9 years) at the time of baseline examination with severe chronic periodontitis [22] participated in this prospective, randomized clinical trial (RCT). The subjects were recruited consecutively from the list of patients seeking periodontal treatment at the Department of Periodontology, Faculty of Dental Medicine, Al-Azhar University, Cairo, Egypt between November 2017 and April 2018. The criteria implemented for patient inclusion were: (a) no systemic diseases that could influence the outcome of therapy; (b) good compliance with plaque control instructions following initial therapy; (c) teeth involved all vital with no mobility [23]; (d) a single, predominately 2- or 3-wall intra-bony interproximal defect around premolar or molar teeth without furcation involvement; (e) selected intra-bony defects (IBD) measured from the alveolar crest to the defect base in diagnostic periapical radiographs of ≥3 mm, with a width of ≥3 mm at its most coronal part, with no cratering, involving both mesial and distal surfaces of adjacent teeth; (f) selected probing depth (PD) ≥6 mm and clinical attachment loss (CAL) ≥4 mm at the site of intraosseous defect four weeks following initial cause-related therapy; (g) intra-bony defects of two or three walls with 45–55-degree angulation were selected in order to reduce defect variability; (h) availability for the follow-up and maintenance program; and (i) absence of periodontal treatment during the previous year. Pregnant females were excluded from participation in the study.

### 2.2. Presurgical Therapy and Grouping

Initial cause-related therapy consisted of thorough full-mouth scaling and root planning performed in quadrants under local anesthesia. This procedure was performed using a combination of hand and ultrasonic instrumentation (Acteon P5, Satalec, Acteon, France) using a P10 tip. Patients were recalled within 24 h to complete initial therapy and to receive detailed mechanical plaque-control instructions. Four weeks following initial therapy, a re-evaluation was performed to confirm periodontal surgery indication. Criteria indicating that surgery was necessary included the persistence of an interproximal site with PD ≥6 mm, CAL ≥4 mm, and interproximal IBD of ≥3 mm. With an acrylic stent in position, baseline periodontal disease status of the selected sites was determined by clinical assessments of plaque index (PI) [24], gingival index (GI) [25], PD [26], and CAL [27]. The deepest point of the selected defect was selected in the calculations. Periapical views using intraoral size 2 dental films (Kodak Extraspeed, Eastman Kodak, Rochester, NY, USA) were recorded using long cone paralleling technique and holders that were guided in a standardized position with the aid of acrylic resin customized bite block with Rinn XCP (Rinn XCP X-Ray Alignment Instruments, DENTSPLY Ltd., Weybridge, UK) employing an x-ray unit (Heliodent 70, Siemens, Bensheim, Germany) operating at 70 kV, 10 mA, and 0.8-second exposure time for IBD measurements. The relative defect base level (DBL) was measured from the cementoenamel junction (CEJ) to the base of the defect, and the relative crestal bone level (CBL) was measured from CEJ to the alveolar crest. Crestal interseptal bone width (CIBW) was measured 1 mm apical to the alveolar crest. Initial cause-related therapy and clinical measurements were performed by an experienced qualified examiner who was not involved in the study in any other way (MTD). Intra-examiner reproducibility was assessed with a calibration exercise performed on two separate occasions, 48 h apart. Calibration was accepted if 90% of the recordings could be reproduced within 1.0 mm.

### 2.3. Randomization and Allocation Concealment

Patients were randomly assigned into one of 2 groups (n = 8): Group 1—bone substitute grafting and Group 2—osseous wall swaging (OWS) combined with xenograft. Computer-assessed randomization was carried out using online software (http://www.randomizer.org) (accessed on 1 January 2023) immediately before surgery. The participants who took part in the study were given a special identification code. The randomization and allocation concealment was carried out by an outside faculty member who was not informed of the study’s objectives. The participants were advised to keep their group identity concealed from the treating clinician.

### 2.4. Surgical Procedures

The surgical treatment phase was initiated only if the subjects had a full-mouth dental plaque score of less than one and a test site plaque score of 0. Following mucoperiosteal flap reflection for one tooth on either side of the defect, all granulation tissue was removed from the defects utilizing Gracey curettes (1/2 and 7/8, Hu-Friedy, Chicago, IL, USA) and root surfaces were scaled and planed using hand and ultrasonic instruments (Acteon P5, Satalec, Mérignac, France), etched with 24% EDTA (Pref Gel, Biora, Malmo, Sweden) for 2 min followed by copious water irrigation. For Group 1, graft substitute (bovine-derived xenograft-Tutongen-RTI/Biologics, Neunkirchen am Brand, Germany) was heavily condensed into the intra-bony defects. For group 2, the proximal osseous wall was split using a piezoelectric knife (Peizotome solo led, Satalec, Acteon, France) under copious water irrigation 1 mm apical to the alveolar crest. Osseous wall splitting was extended apically up to the level of the defect base. The split bony wall was tilted toward the periodontitis-affected exposed root surface using the sequential size of hand expanders (Surgident Co., Ltd., Daegu, Republic of Korea) until it was 1 mm away from the periodontally affected exposed root surface. Care was taken not to induce extra force that could fracture the base of the split bony wall, especially for lower teeth with mostly compact bone at the most coronal third. The created V-shaped gap between adjacent-tooth bony wall and swaged bone was filled tightly with the graft substitute. Finally, the flap was replaced and closed with crossed horizontal and vertical internal mattress sutures.

### 2.5. Postoperative Care

All patients received oral and written postoperative instructions. Patients were prescribed amoxicillin (500 mg, October Pharma, Cairo, Egypt) every 8 h for 1 week. Plaque-control effort was supplemented by rinsing with 0.12% chlorhexidine hydrochloride (Surgident Co., Ltd., Daegu, Republic of Korea) for one minute 2 times daily for 2 weeks. Patients were instructed to refrain from brushing and interdental cleaning at the surgical areas during this time.

### 2.6. Follow-Up and Re-Evaluation

Sutures were removed 2 weeks postoperatively and recall appointments for observation of any adverse tissue reaction and oral hygiene reinforcement were scheduled every second week during the first 2 months after surgery. Three weeks after surgery, all patients were instructed to resume their normal mechanical oral hygiene measures, which comprised brushing using a soft toothbrush with a roll technique and flossing. Supportive periodontal maintenance, including oral hygiene reinforcement and supragingival scaling, were performed during each recall appointment.

### 2.7. Post-Surgical Measurements

Clinical and radiographic evaluations were performed 6 months after the surgery. The evaluation period was in accordance with those in previous studies [28,29].

### 2.8. Primary and Secondary Outcome Measures

The primary outcome of this clinical trial was bone-defect fill as evaluated by the radiographs, and the secondary outcomes included clinical parameter evaluation of PD, CAL, GI, and PI.

### 2.9. Statistical Analysis

Statistical analysis was performed with statistical software (NCSS-PASS^®^, Number Cruncher Statistical Systems, Kaysville, UT, USA). The sample size was calculated using the PASS^®^ v.11 program, setting the type-1 error (α) at 0.05 and power at 80%. Results from a previous study [11] showed that the intra-bony defect in the treatment group was 1.9 ± 0.5 compared to 2.9 ± 0.5 in the control group. Calculation according to these values showed a minimal sample size of 6 cases per study group; however, taking into consideration of a 20% dropout rate, 8 cases were included per group. Data distribution was normal and, hence, parametric test was applied (Shapiro–Wilk test; *p* > 0.05). Periodontal status data were presented as mean ± SD values. Paired t-test was used to compare the control and experimental sites within each group, as well as to analyze the changes by time within each group. The significance level was set at *p* ≤ 0.05.

## 3. Results

**Figure 1:**
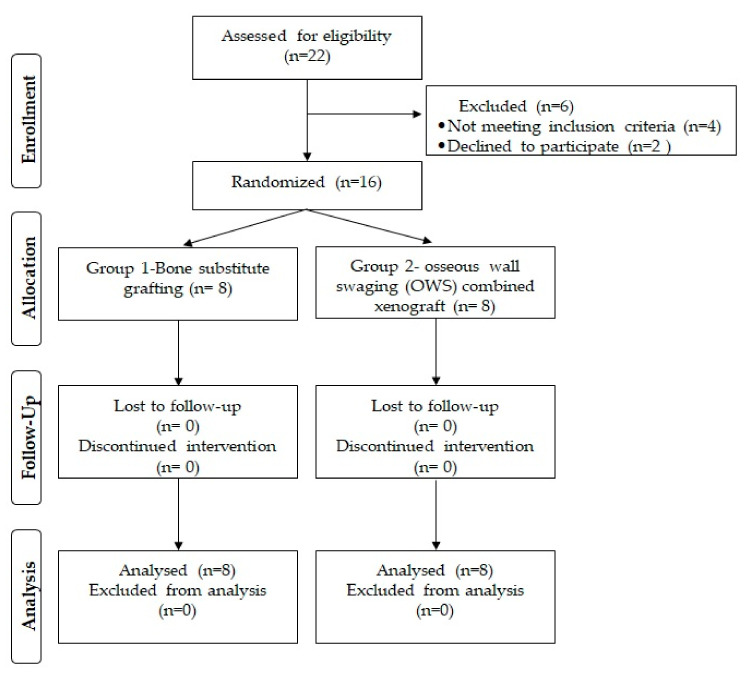
CONSORT flow chart of the clinical trial.

In total, twenty-two patients were assessed for eligibility and six of them were excluded. Of the excluded patients, four did not meet the inclusion criteria and two declined to participate in the study. Finally, the 16 patients enrolled in this trial were randomly allocated into two groups. None of the patients in either of the group were lost to follow-up and all the enrolled patients’ data were available for final analysis. Figure 1 presents the CONSORT flow chart of the clinical trial detailing the study process.

Group 1 consisted of two maxillary premolars, one maxillary first molar, one mandibular premolar, and four mandibular molars. Group 2 consisted of two maxillary premolars, two maxillary first molar, two mandibular premolars, and two mandibular first molar. The treated defects were distributed as follows: for the Group 1 sites, five predominately two-wall and three predominately three-wall defects; for the Group 2 sites, four predominately two-wall and four predominately three-wall defects. Table 1 shows the individual defect location and anatomic characteristics. All the patients showed good compliance, with uneventful healing following therapy and good soft-tissue responses to both treatment options. The patients in both groups exhibited comparable oral hygiene standards and continued their clinical follow-up visits. No site had to be eliminated, and no sites of flap dehiscence or unwanted infection were detected. Minimal swelling of the soft tissues at the operated areas was observed during the early days of post-operative healing.

The periodontal status (PI, GI, PD, CAL, CBL, DBL, and CIBW) comparison of the two groups at baseline and after surgery is presented as mean ± SD (Table 2). No statistically significant differences were found preoperatively between both groups regarding the soft- and hard-tissue measurements (*p* > 0.05). All the GI and PI scores were within clinically healthy limits. The selected teeth were free of gingival inflammation and plaque before surgery and at the end of the study. The patients were kept under a meticulous maintenance program, and the overall plaque accumulation was minimal.

The comparisons by time for the control Group 1 and Group 2 OWS groups revealed a significant reduction in PD and CAL (baseline versus 6 months) for both groups (*p* > 0.05). The DBL was significantly reduced compared to baseline for both groups at the 6-month observation periods. For Group 1, the postoperative CBL at 6 months was reduced by 0.9 mm, which was statistically lower than that of the baseline value. The Group 2 patients treated by OWS showed a statistically significant gain in CBL at 6 months compared to the baseline values. The comparison between groups revealed that, at the 6-month observation period, the OWS-treated sites showed a statistically significant improvement in PD reduction compared with Group 1. The CAL was statistically higher for the OWS compared with that of the Group-1-treated sites at the 6-month observation periods (*p* > 0.001). The DBL was significantly reduced for the OWS group compared to Group 1 at the 6-month observation period. The CBL was significantly higher in the OWS group compared to Group 1 at the 6-months observation periods (*p* > 0.001). The crestal interseptal bone width (CIBW), as measured one millimeter apical to the alveolar crest, was found to be non-significantly affected in the Group 1 control when compared to the 6-month value. However, it was found to be significantly higher in Group 2 at 6 months compared to the baseline value and to that of Group 1.

**Figure 2:**
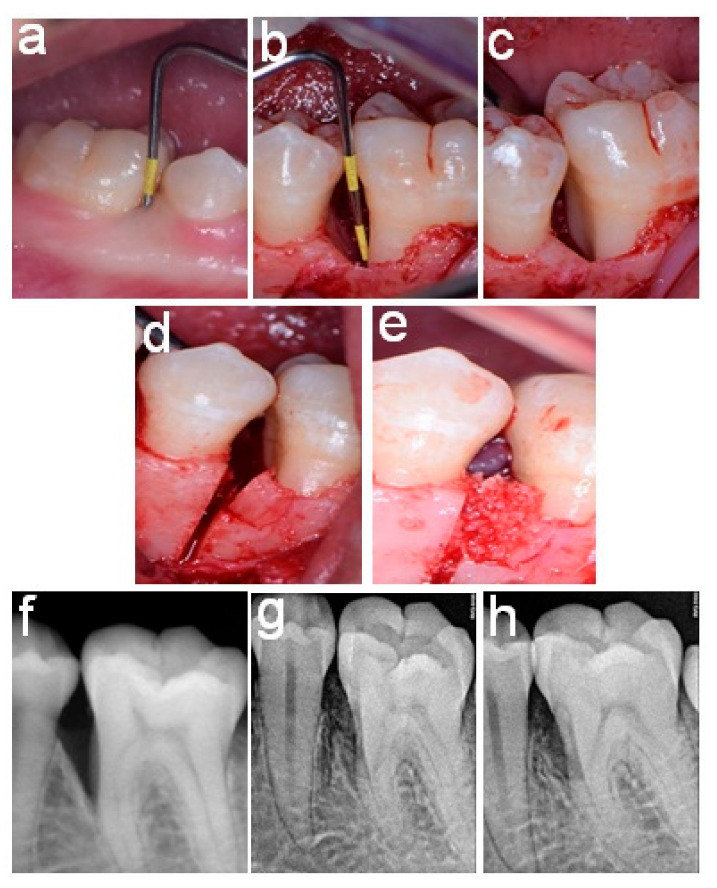
An 8-millimeter pocket related to the mesial surface of lower-left first molar of Group 1 patient. (**a**) Intra-bony defect of 5 mm, (**b**,**c**) osseous wall cutting and tilting, (**d**,**e**) grafting of the V-shaped space and (**f**–**h**) intra-oral radiograph obtained before, immediately after, and 6 months after splitting.

**Figure 3:**
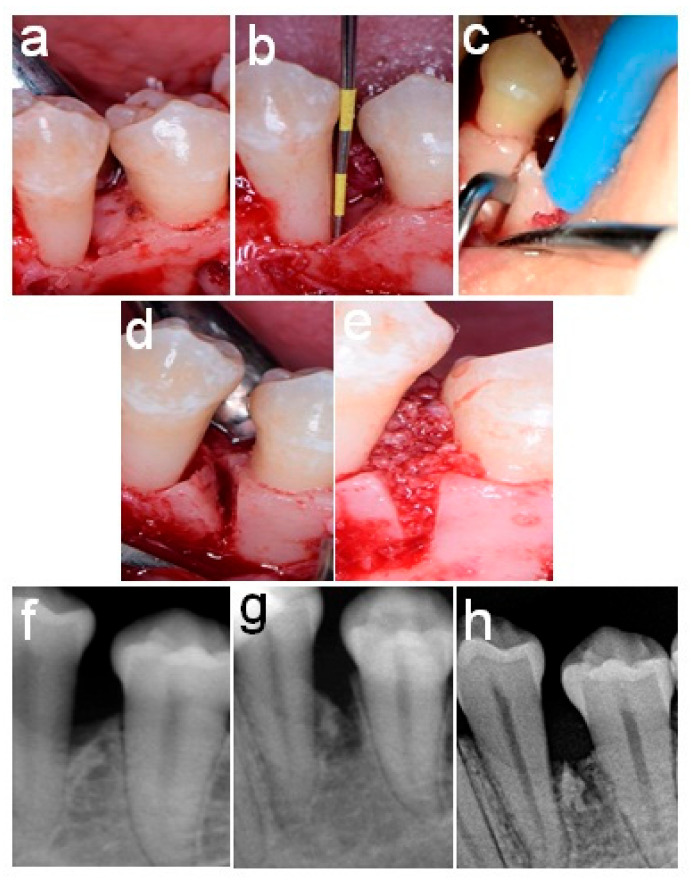
A 6-millimeter-wide two-wall intra-bony defect related to the distal surface of lower-left first premolar of a Group 2 patient. (**a**–**c**) Osseous wall cutting and tilting (**d**,**e**) grafting of the V-shaped space, and (**f**–**h**) intra-oral radiograph obtained before, immediately after, and 6 months after splitting.

Figure 2 and Figure 3 present the pre-and post-surgical intra-oral patient photographs of patients from Group 1 and Group 2, respectively.

## 4. Discussion

To the best of our knowledge, this is the first clinical trial to have employed minimally invasive piezo-cutting of a vascularized bony wall combined with bone-expander wall redirection and EDTA root surface treatment when dealing with intra-bony defects with normal interdental space dimensions and wide angulations. The outcome of the current study demonstrated that the applied protocol allowed the use of osseous wall swaging at any interdental space dimensions with minimal risk of cortical bone base fracture.

The periodontal osseous wall swaging in all of the limited studies available [18,19,20,21,30] required the presence of an edentulous area adjacent to the defect, which limited its use and was very uncommon. In the present study, we utilized a piezoelectric knife [31] with micrometric cut and variable forms of micro tips, which provided safe and precise osteotomies without any reported osteonecrosis at the interproximal bony walls in teeth with tight contact and narrow bony walls. By contrast, conventional macro piezoelectric knives were used by Shirakata et al. [30] who performed bone swaging after creating an edentulous area adjacent to an experimental one-wall defect to ensure accessibility. Furthermore, piezocesion works only on mineralized tissues, sparing soft tissues and their blood supply, a factor that ensures grafted bone viability [31]. Bone swaging also requires bone elasticity to provide a continuous bone graft. Piezocutting and the sequential use of bone expanders used in this study instead of the bone chisel used in other studies was found to ensure maximum protection for the grafted pedicle, especially mandibular teeth, without an adequate cancellous composition. The healing of the grafted V-shaped space that was created following bony wall tilting toward the root surface appeared to be less challenging. The border around the space was converted from a avascular smear coated root surface on one side and the bony wall of the pocket on the other to two highly vascular and cellular bony walls, with an associated maintenance of the blood supply and improvements in osteogenesis from the contiguous vital bone. The xenogeneic grafting of such an area was reported to stabilize the grafted swaged bone and prevent micromovements that may increase the resorption rate of the graft following transplantation [32].

The comparison between the groups revealed that, at the 6-month observation periods, the OWS-treated sites showed a statistically significant improvement in PD reduction and CAL compared to the control Group 1 patients. This could be explained by the higher bony support that was provided by the split bone. In addition, the EDTA root surface etching and the reduced defect angulation that followed the osseous wall splitting led to enhanced clot-root surface adsorption and significantly reduced intra-defect clot retraction, factors that can improve pocket reduction and attachment gain [33,34]. Bone grafting as a single therapy was found in many studies to offer non-significant outcomes compared to open flap debridement, possibly because of the marked clot-blended graft retraction, especially with wide defect angulations. Many histological analyses revealed no or only an unpredictable amount of periodontal regeneration following the application of alloplastic materials as a single therapy [35,36]. The results of this study were similar to those of a study performed by Kodama et al. [20], who evaluated the effectiveness of guided tissue regeneration using a resorbable collagen membrane and bone swaging in non-contained infra-bony defects and reported improved PD, CAL, and radiographic bone fill 2 years after therapy. The main difference between their study and ours is their use of fissure burs and a bone chisel for bone swaging. In addition, Kodama et al. limited the study cases to those with ≥4mm of interdental space, contrary to the present study, in which cases with normal interdental spaces were included.

The DBL was significantly reduced for the OWS group compared to that of Group 1 at the 6-month observation period. The reduced defect angulation offered by OWS maintained the suggested EDTA root surface etching with more clot-blended graft stability and reduced clot thickness, with an associated augmented defect base bone filling. Gamal [37] reported that root conditioning with EDTA gel improved grafted particle adhesion to periodontally involved root surfaces. The author attributed this improved adhesion to the incorporation of clotted blood and graft materials within the EDTA-opened and -widened dentinal tubules. The non-resorbable nature of the used xenogeneic graft material for Group 1 compared to the lack of defect grafting for Group 2 indicate the healing by the radiopaque bony material at the defect base for the OWS group. At the same time, the CBL was found to be significantly higher in the OWS group compared with that of Group 1 at the 6-month observation periods.

The vascularized bone pedicle allowed significantly increased vascular supply and, subsequently, reduced post-operative crestal resorption for the split bony wall. These findings support the experimental data reported by Shirakata et al. [30], who evaluated bone swaging and Emdogain over the root surface together with injectable calcium phosphate bone cement. They reported 3.68 ± 0.33 mm of newly formed bone, 2.74 ± 0.33 mm of crestal bone gain, and cementum formation of 3.93 ± 0.56 mm following osseous one-wall swaging. Furthermore, Shirakata et al. [38] evaluated histologically the newly formed tissue following bone swaging in dogs. They reported new bone formation from the host bone toward the coronal region of the defects in the bone swaging group. However, Zubery et al. [21], in a case report, found no evidence of the formation of new cementum with organized periodontal ligament fibers between the root surface and the separated swaged bone graft. The findings of the present study were similar to those of other studies, which showed that the use of autogenous bone grafts induced the complete integration of the bone grafts into new trabecular bone formed at the recipient site [39,40,41]. The CIBW was found to be significantly higher in Group 2 at 6 months compared to the baseline value and to that of Group 1 during the 6-month observation period. This finding could suggest improved resistance to crestal bone resorption following the use of OWS through improved crestal bone thickness.

This clinical trial was not without limitations. Firstly, the limited sample size made it possible to classify the selected defects based on tooth type or location. Secondly, the outcome was evaluated for a short-term period of 6 months. Further studies are needed to evaluate the stability of the clinical data on a long-term basis. The improved crestal bone thickness reported in the present study appears to be an important finding that needs further investigation due to its potential importance in enhanced resistance to future alveolar bone loss. Different piezo-tips that suit the cutting of interdental bone with tight contact and thin dimensions should be developed to render osseous wall swaging much more familiar.

## 5. Conclusions

Within the limitations of this clinical trial, it was concluded that OWS effectively improved the clinical hard- and soft-tissue parameters. The use of mini-insert piezocutting, sequential bone expanders for osseous wall redirection, and root surface EDTA etching appeared to be a reliable approach that could allow the use of OWS at any interproximal dimension.

## Figures and Tables

**Table 1:** Individual defect location and anatomic characteristics.

Patient No.	Group 1			Group 2		
	Tooth Number and Surface	Defect Type	Bony Walls Present	Tooth Number and Surface	Defect Type	Bony Walls Present
1	5 M	2-Wall	DL	5 M	2-Wall	BLD
2	4 M	3-Wall	BLD	13 M	2-Wall	BL
3	3 M	3-Wall	BLD	14 D	3-Wall	BLD
4	20 D	2-Wall	BL	14 M	2-Wall	BM
5	19 M	2-Wall	BD	28 M	3-Wall	BLD
6	30 M	3-Wall	BLD	29 D	3-Wall	BLD
7	30 M	2-Wall	MDL	30 D	3-Wall	BLD
8	31 D	2-Wall	BD	21 D	2-Wall	BD

D = Distal; B = Buccal; L = Lingual; M = Mesial.

**Table 2:** Mean comparison of clinical and radiographic periodontal parameters between baseline and follow-up measurement at six months.

Parameter	Group 1			Group 2		
	Baseline	6 Months	*p*-Value	Baseline	6 Months	*p*-Value
**Clinical parameters**						
PD (mm)	6.2 ± 0.3 ^a^	3.6 ± 0.4 ^b^	<0.001 *	5.9 ± 0.6 ^a^	2.4 ± 0.4 ^b^	<0.001 *
CAL (mm)	5.7 ± 0.5 ^a^	3.7 ± 0.5 ^b^	<0.001 *	5.1 ± 0.4 ^a^	2.2 ± 0.3 ^b^	<0.001 *
PI	0.4 ± 0.1	0.3 ± 0.2	0.427	0.5 ± 0.2	0.5 ± 0.2	0.233
GI	0.4 ± 03	0.5 ± 0.2	0.521	0.4 ± 0.2	0.3 ± 0.3	0.239
**Radiographic parameters**						
DBL (mm)	8.7 ± 0.6 ^a^	6.7 ± 1 ^b^	<0.001 *	9.2 ± 0.7 ^a^	3.1 ± 0.4 ^b^	<0.001 *
CBL (mm)	5.6 ± 0.7 ^b^	6.5 ± 1.2 ^a^	0.02 *	5.8 ± 0.8 ^a^	3.2 ± 0.3 ^b^	<0.001 *
CIBW	1.2 ± 0.3	1.4 ± 0.1	0.233	1.4 ± 0.3 ^a^	3.4 ± 0.4 ^b^	<0.001 *

PD–probing depth; CAL–clinical attachment Loss; PI–periodontal index; GI–gingival index; DBL–defect base level; CBL–crestal bone level; CIBW–crestal interseptal bone width. * Significant at *p* ≤ 0.05. Different lower case in the same row indicates significant differences in periodontal parameters from baseline to 6 months post-treatment.

## Data Availability

The data presented in this study are available upon request from the corresponding author.
